# Impact of Lipid Composition and Synergistic Effect on Aβ42 Oligomer-Induced Changes in Membrane Conductance and Microviscosity

**DOI:** 10.1021/acschemneuro.6c00244

**Published:** 2026-08-31

**Authors:** Evelina Jankaitytė, Artu̅ras Polita, Andrius Sakalauskas, Rimgailė Tamulytė, Vytautas Smirnovas, Rima Budvytyte

**Affiliations:** † Institute of Biochemistry, Life Sciences Center, 54694 Vilnius University, Saulėtekio av. 7, Vilnius LT-10257, Lithuania; ‡ Institute of Biotechnology, Life Sciences Center, Vilnius University, Saulėtekio av. 7, Vilnius LT-10257, Lithuania

**Keywords:** β-amyloid, Aβ42, aggregation, lipid membranes, membrane damage, microviscosity, permeability

## Abstract

The accumulation of the Aβ42 peptide is one of the key factors in the progression of Alzheimer’s disease, as it disrupts synaptic signaling and impairs neuronal function. Although the peptide is known to compromise plasma membrane integrity, the detailed mechanisms underlying bilayer alterations and lipid–Aβ42 interactions remain unclear. In the present study, electrochemical impedance spectroscopy and fluorescence lifetime imaging microscopy were employed to investigate the impact of oligomeric Aβ42 on model membranes that mimic the lipid composition of the outer leaflet of the neuronal plasma membrane. Our results demonstrate that the presence of SM or GM1 within a DOPC/Chol backbone is enough to facilitate Aβ42 interaction with the membrane, as evidenced by increased membrane microviscosity; however, this interaction alone is insufficient to induce permeability-enhancing defects in the membrane. Extensive disruption of bilayer integrity required the complete lipid ensemble present in the outer leaflet of the neuronal membrane-mimicking composition, indicating that not only specific lipids but also their cooperative interplay and organization within the lipid bilayer are essential for Aβ42-induced membrane permeabilization.

## Graphical abstract

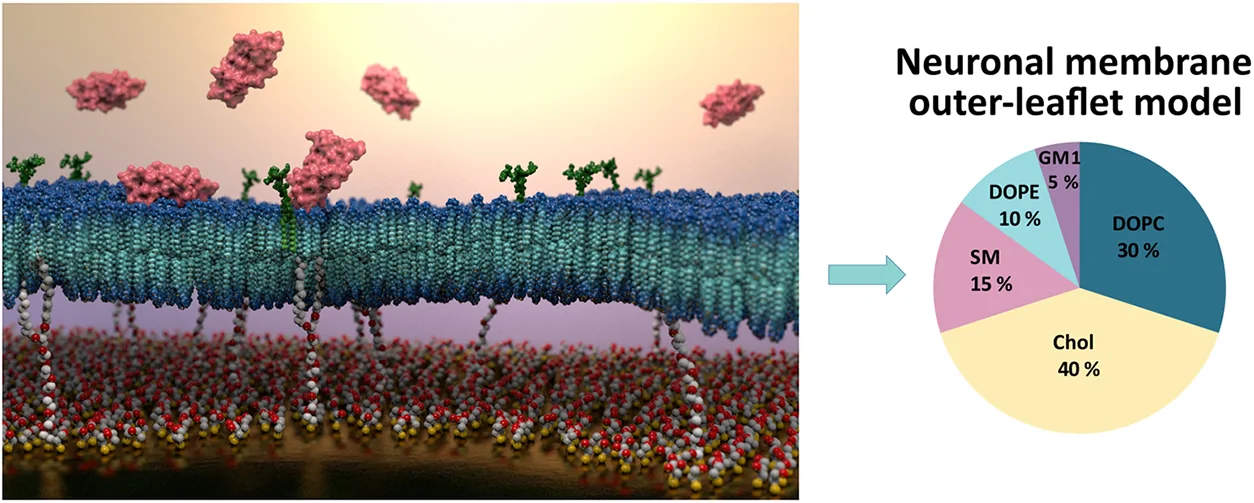

## 1 Introduction

Under certain pathological conditions, disease-related proteins can undergo structural rearrangements, adopting misfolded conformations that aggregate into highly ordered amyloid assemblies rich in cross-β structures.^1^ The decline in proteostasis during aging, resulting from reduced chaperone activity, impaired proteasomal and autophagic degradation, and elevated oxidative stress, promotes the accumulation of misfolded proteins in the brain, leading to the formation of amyloid plaques.^2,3^ Alzheimer’s disease (AD), the most prevalent neurodegenerative disorder and the leading cause of dementia, is characterized by the extracellular deposition of amyloid-β (Aβ) peptide in the brain.^4^ Among the Aβ isoforms, the 42-residue variant (Aβ42) exhibits the highest aggregation propensity and is associated with amyloid plaque formation and neuronal toxicity.^5,6^ Soluble Aβ42 oligomers are now recognized as the principal toxic species, capable of impairing synaptic function and neuronal viability before fibrillar deposits appear.^7,8^ Growing evidence indicates that these oligomers can exert deleterious effects through interactions with neuronal membranes, leading to ionic imbalance, oxidative stress, and synaptic dysfunction.^4,9–11^

Lipids such as gangliosides (GM), cholesterol (Chol), and brain sphingomyelin (SM) are widely recognized as key modulators of Aβ aggregation and fibrillation.^12,13^ While single lipid species can influence Aβ aggregation, *in vivo* biological membranes present these components within a heterogeneous, multicomponent environment. Consequently, the overall impact of lipid composition in mixed, physiologically relevant bilayers remains difficult to resolve. Beyond its aggregation propensity, Aβ can also impair neuronal conductivity by altering ion channel function. *In vivo* studies have demonstrated that Aβ42 interacts with L-type Ca^2+^ channels, resulting in excessive calcium influx and subsequent membrane depolarization in hypothalamic neurons.^14^ While Aβ42 is known to perturb lipid bilayer organization, its influence on membrane fluidity remains controversial, with both increases and decreases reported under varying experimental conditions. For example, Madhu et al. demonstrated that certain Aβ42 oligomers undergo a conformational change upon binding to the membrane, allowing deeper insertion into the lipid bilayer thereby increasing microviscosity.^15^ In contrast, Kubánkova et al. found that oligomeric Aβ42 decreased plasma membrane microviscosity in live SH-SY5Y and HeLa cells, indicating increased fluidity.^16^ Nevertheless, despite extensive research, the molecular mechanisms underlying Aβ42-induced cytotoxicity remain incompletely understood.

To gain molecular-level insight into the mechanism underlying Aβ42-induced membrane alterations, we employed electrochemical impedance spectroscopy (EIS) in combination with fluorescence lifetime imaging microscopy (FLIM) using a viscosity-sensitive dye to characterize the physical properties of tethered bilayer lipid membranes (tBLM). tBLMs are solid-supported model lipid bilayers that provide a well-defined and stable platform for probing protein–lipid interactions. Their architecture makes them compatible with surface-sensitive techniques, allowing precise investigation of membrane-associated processes.^17^

In this study, we prepared a tBLM mimicking the neuronal outer leaflet, composed of dioleoyl-phosphocholine (DOPC), Chol, SM, dioleoyl-phosphoethanolamine (DOPE), and GM1.^18,19^ With DOPC and Chol as the base composition, we evaluated the contributions of SM, GM1, and DOPE. These models enabled us to probe Aβ42 interactions with biologically relevant lipids and assess their impact on membrane dynamics and integrity upon interaction with the peptide. Through the mathematical modeling of EIS spectra, we quantitatively assessed the density and heterogeneity of Aβ42-induced defects within the tBLMs. This integrative approach provides in-depth insight into how distinct lipid environments modulate Aβ42-mediated membrane disruption, offering a mechanistic understanding of lipid composition-dependent effects.

## 2 Results and Discussion

### 2.1 Aggregation of Aβ42

To characterize recombinant Aβ42, its aggregation kinetics were monitored using the thioflavin T (ThT) fluorescence assay, which detects fibril formation through enhanced fluorescence upon binding to β-sheet-rich structures.^20^ Measurements were performed at monomer concentrations ranging from 0.5 to 2 μM. The resulting sigmoidal curves of Aβ42 aggregation reflects its multistep process,^21^ with higher peptide concentrations leading to a shorter lag time (Figure 1A). During the initial lag phase, soluble monomers self-assemble to form oligomers.^22^ As this study focuses on Aβ42 oligomer-membrane interactions, all subsequent experiments were performed using peptides from the aggregation stage before the end of the lag phase.

**Figure 1:**
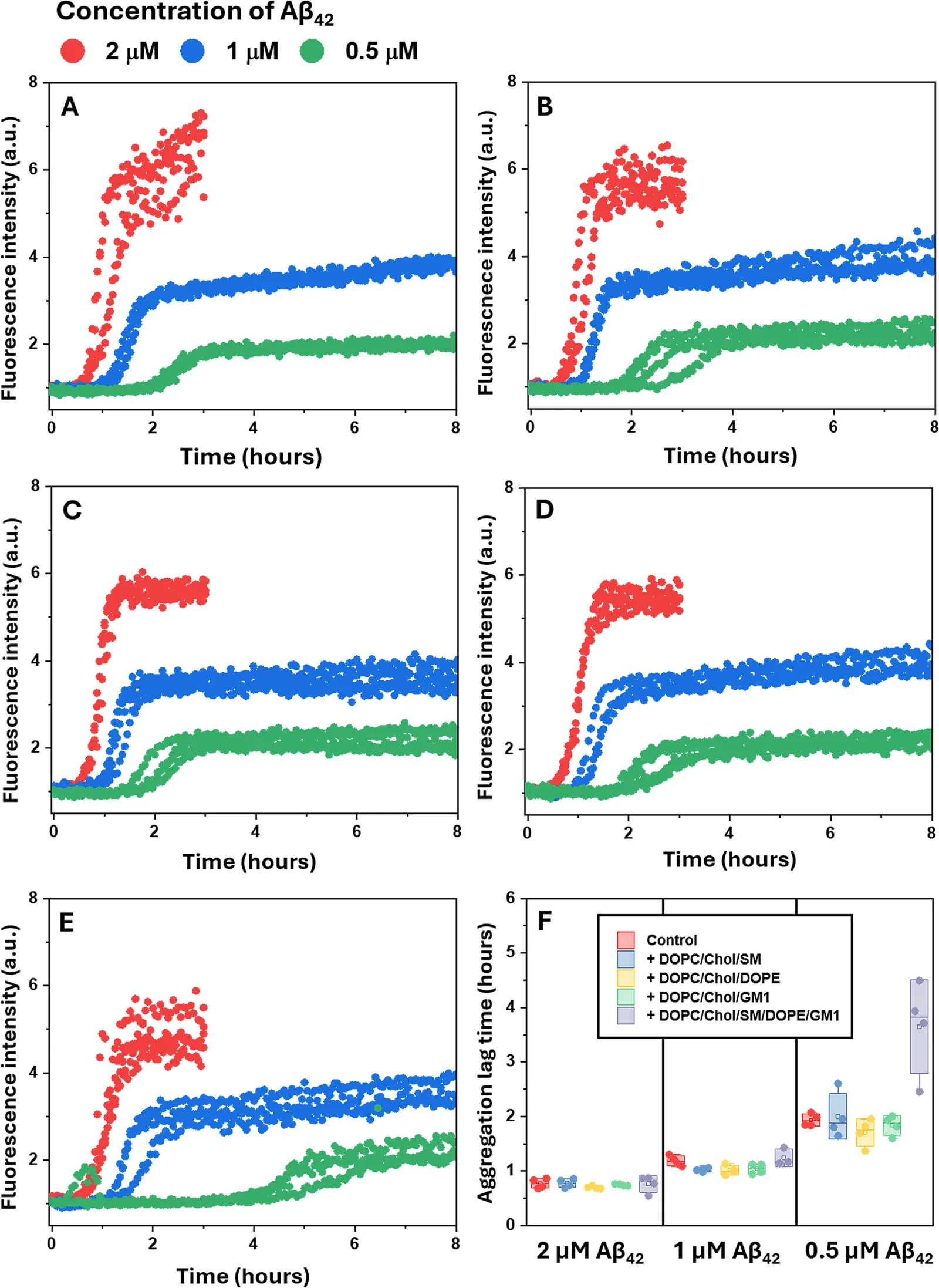
ThT fluorescence kinetics of Aβ42 aggregation at 2, 1, and 0.5 μM measured in the (A) absence (control) and in the presence of liposomes with different lipid compositions: (B) DOPC/Chol/SM (45/40/15), (C) DOPC/Chol/DOPE (50/40/10), (D) DOPC/Chol/GM1 (55/40/5), and (E) DOPC/Chol/SM/DOPE/GM1 (30/40/15/10/5). (F) Box plot summarizing the corresponding aggregation lag times. Statistical significance was assessed by comparing lag times measured with each liposome formulation to the no-liposome control (Aβ42 alone). *p* < 0.05 was considered significant; *** (*p* < 0.001).

### 2.2 Effect of Lipid Composition on Aβ42 Aggregation

Aβ42 is an amphiphilic peptide capable of interacting with a variety of lipids, and these interactions can significantly influence its aggregation pathways. Adsorption of Aβ42 to the membrane surface may exert a reciprocal effect, not only modulating fibrillization but also compromising membrane integrity.^23,24^ To investigate the influence of lipid bilayer compositions on Aβ42 aggregation, we used large unilamellar vesicles (LUVs) composed of different lipid mixtures; corresponding hydrodynamic diameters are provided in the Supporting Information (Table S1). In most cases, LUVs did not markedly alter Aβ42 aggregation kinetics (Figure 1A–E) or lag time (Figure 1F). However, at lower peptide concentration (0.5 μM), five-component LUVs decelerated aggregation of Aβ42 (Figure 1E,F). Although lipid membranes are often reported to accelerate Aβ42 aggregation, this effect is not universal.^24–26^ Differences between published results may therefore arise from variations in membrane composition and physicochemical properties, peptide-to-lipid ratio, and experimental conditions. Sanguanini et al. showed that individual lipid components can either enhance or inhibit Aβ42 aggregation. In contrast, mixtures of multiple lipids tend to buffer these extreme effects, producing a “resilience in complexity” against aggregation.^27^ Similarly, Baumann et al. demonstrated that biomimetic membrane compositions corresponding to different cellular locations have distinct effects on Aβ42 aggregation, with ER and Golgi model membranes inhibiting fibril formation.^28^ In this context, the delayed aggregation observed in the presence of the five-component LUVs (Figure 1E) may arise from a composition-dependent buffering effect of the complex lipid mixture. Cooperative interactions among GM1, SM, DOPE, and Chol within the DOPC matrix may generate a heterogeneous membrane surface that favors Aβ42 adsorption while limiting productive fibril nucleation. Such synergy is consistent with known interactions among raft-associated lipids; for example, cholesterol can modulate glycosphingolipid conformation, thereby affecting Aβ40 binding.^29^ Moreover, cholesterol itself has been reported to exert context-dependent effects on Aβ-membrane interactions. In some systems, cholesterol promotes Aβ42 surface aggregation, whereas in others it limits peptide penetration into the bilayer and reduces membrane disruption.^30^ These findings further support the idea that the effect of an individual lipid component depends strongly on the surrounding overall membrane composition and environment.

At higher Aβ42 concentrations (1–2 μM), the inhibitory effect of the five-component LUVs was no longer evident. This may be due to the saturation of the membrane surface, after which excess peptide remains in solution and undergoes self-association, thereby reducing the apparent lipid-dependent effect on aggregation. Importantly, although soluble oligomeric Aβ species are widely considered among the most toxic forms of the peptide,^7,31^ membrane damage may arise during the aggregation process and amyloid growth.^32,33^ Therefore, the delayed fibrillization observed in the five-component system may be relevant because it extends the time window during which membrane-bound aggregation intermediates interact with and destabilize the lipid bilayer.

### 2.3 Morphological Characterization of Aβ42 by Atomic Force Microscopy (AFM)

To verify the presence of oligomeric Aβ42 , we investigated the Aβ42 morphology using AFM (Figure 2A,C). Samples were taken at the onset of peptide aggregation and after 30 min. The results indicated no alterations in oligomer morphology and no significant increase in average height. The apparent lack of structural differences may not necessarily reflect completely unchanged peptide morphology but could instead arise from the drying of Aβ42 samples on APTES-functionalized mica, where adsorption and surface-induced rearrangements can mask early time variations and render the structures deceptively similar. Aliquots taken at different time points during the aggregation process, corresponding to the time window used for subsequent experiments, yielded a consistent average particle size of 1.6 ± 0.5 nm. The height values for the initial samples ranged from 0.5 to 3.5 nm, whereas the Aβ42 oligomers collected at 30 min exhibited a distribution ranging from 1.0 to 3.5 nm (Figure 2B,D). This indicates that Aβ42 was investigated at a prefibrillar oligomeric stage, prior to the end of the lag phase.

**Figure 2:**
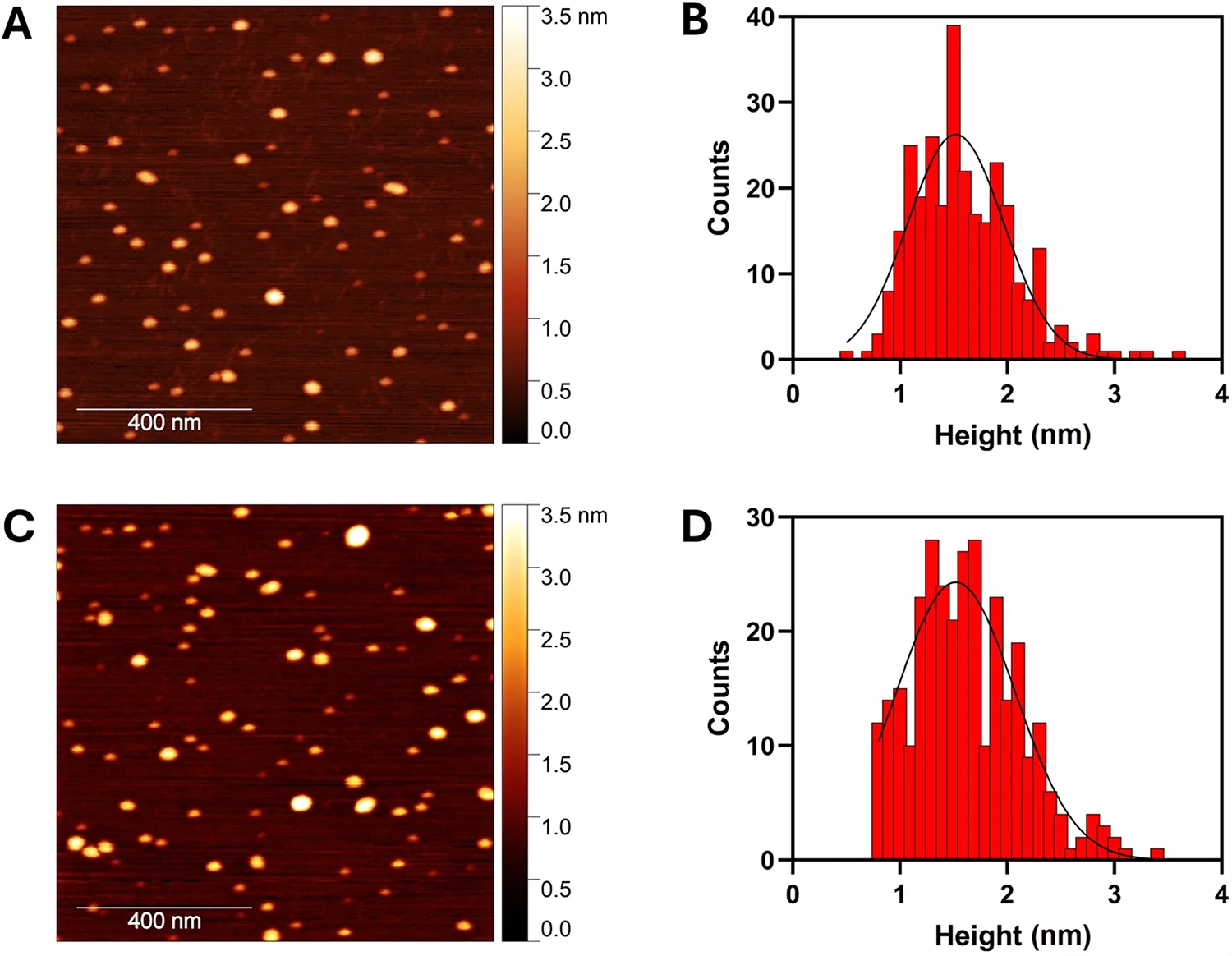
AFM topography of Aβ42 oligomers (2 μM) (A) at the onset and (C) after 30 min of aggregation, and (B, D) the corresponding size distribution histograms.

### 2.4 The Impact of Membrane Composition on Aβ42-Induced Permeability Changes

Protein-induced damage and disruption of membrane integrity in tBLMs can be detected using EIS.^31,34,35^ Defect formation in the tBLM compromises its barrier properties, increasing ionic conductance and altering the measured impedance.^36^

The physical properties of tBLMs with varying lipid compositions were investigated in the presence of Aβ42 oligomers. The formation and stability of tBLMs over the course of the experiments are confirmed in the Supporting Information (Figures S1, S2). The minimum evidently detectable amyloid concentration was 2 μM, as determined through a series of EIS measurements that monitored the corresponding changes in the impedance spectra (Figure S3). EIS spectral changes reveal Aβ42 oligomer-induced variations in tBLM admittance.^36^ The resulting increase in membrane permeability is quantified by the change in admittance *Y* = 1/*Z*_fmin_, evaluated at the minimum point (*f*_min_) of the admittance phase (arg*Y*).^37^ In our case, significant alterations in the EIS spectra were observed only in the neuronal membrane-mimicking tBLM upon exposure to Aβ42 oligomers (Figures 3 and S4). During incubation with Aβ42, a step-like kink developed on a *Y* vs frequency plot (Figure 3A, upper panel). In addition, *f*_min_ shifted toward higher frequencies with increasing Aβ42 incubation time (Figure 3A, bottom panel), indicating membrane damage and altered ion transport across it.^37^ This spectral change corresponds to a substantial increase in admittance, with membrane conductance increasing by 26-fold within 30 min of Aβ42 exposure. After 60 min of incubation with Aβ42, the spectra no longer exhibited the characteristic phase minimum, suggesting a loss of the membrane’s insulating properties. This implies that the damage had advanced to a degree where reliable evaluation of the bilayer’s dielectric behavior by EIS was no longer possible. In contrast, no major changes were observed in the conductance of tBLMs composed of DOPC/Chol/GM1, DOPC/Chol/SM, and DOPC/Chol/DOPE upon exposure to Aβ42 oligomers (Figures 3B and S4).

**Figure 3:**
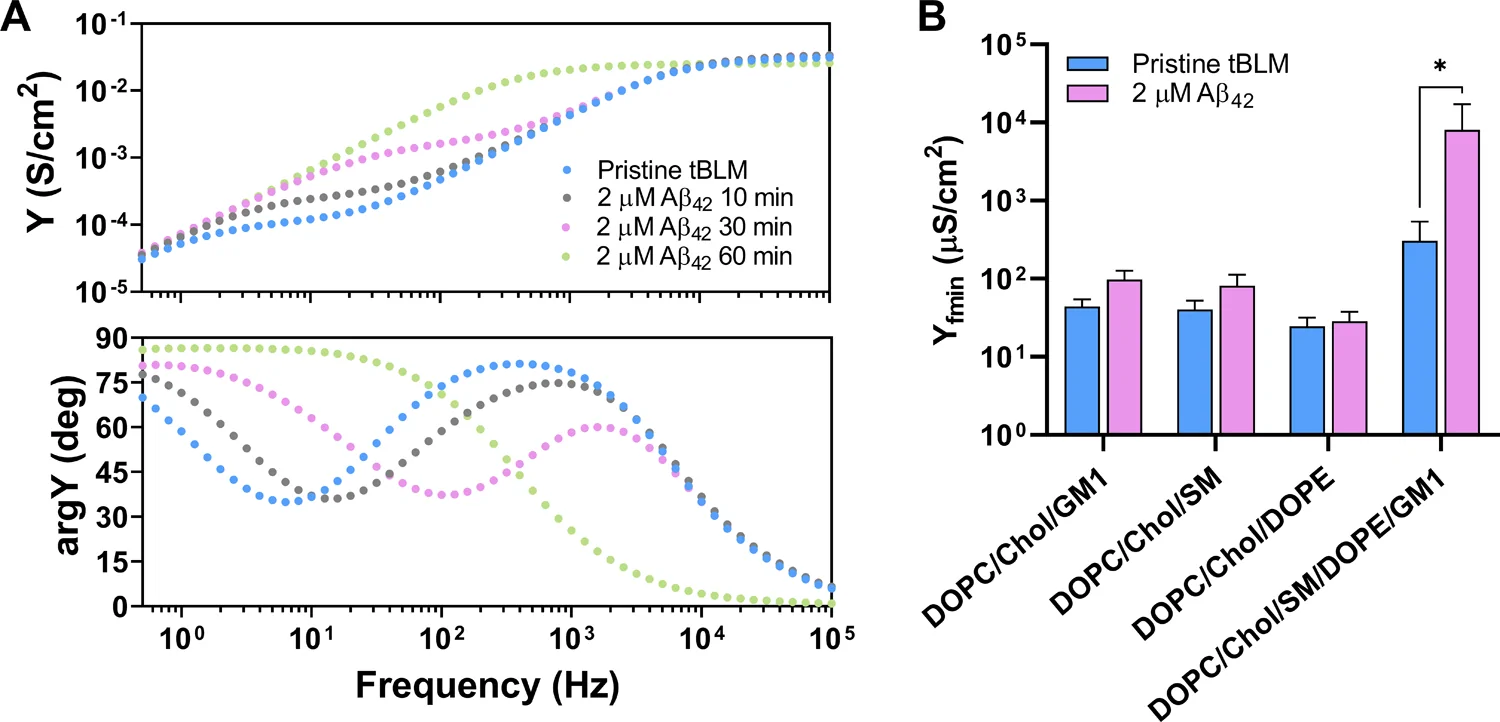
(A) EIS spectral changes in a neuronal outer leaflet-mimicking tBLM (DOPC/Chol/SM/DOPE/GM1 (30/40/15/10/5)) upon exposure to 2 μM Aβ42. The upper panel of the graph displays a plot of *Y* vs frequency, while the lower panel presents a plot of arg*Y* vs frequency. (B) Admittance values of different tBLM systems before and after 30 min incubation with Aβ42 oligomers. Statistical significance is indicated by * (*p* < 0.05).

These findings suggest that Aβ42 oligomer-induced membrane permeabilization is highly dependent on lipid membrane composition, with the greatest degree of permeabilization observed in the neuronal membrane-mimicking tBLM. Similar effects have been reported in planar lipid bilayers, where soluble amyloid oligomers increased bilayer conductance, indicating that oligomeric Aβ species can compromise the dielectric barrier of the membrane and promote ion permeability.^38,39^ Our results are also consistent with the study by Mrdenovic et al., who examined the interactions of Alzheimer’s disease-related Aβ species with floating bilayer lipid membranes containing a comparable phospholipid headgroup classes. They reported that Aβ42 disrupts lipid acyl-chain ordering, whereas oligomers affect lipid headgroup orientation without substantially altering membrane hydration. Notably, oligomeric Aβ induced membrane poration and substantial changes in bilayer electrical properties, supporting the idea that molecular-scale perturbations of lipid organization can translate into functional membrane damage.^40^

### 2.5 Quantitative Evaluation of Defect Density from EIS Spectra

Through mathematical modeling of EIS data, tBLMs can provide not only electrical parameters but also insights into the spatial distribution and density of membrane defects. The computational methodology developed by Ambrulevičius and Valinčius^34^ enables the derivation of the defect-density distribution function, which reveals the homogeneity or heterogeneity of lateral populations of amyloid-induced defects in lipid membranes. The resulting function represents the probability of encountering a surface patch with a given local defect density (*N*_def_). Representative fitted spectra for all lipid compositions are presented in Figure S5. The model-derived curves shown in Figure 4 reveal that pristine membranes exhibit a single-peak defect-density distribution, indicating naturally occurring, monodisperse defects. In the cases of membranes containing DOPC and Chol combined with GM1, SM, or DOPE, changes in defect densities were negligible (Figure 4A–C). However, an apparent effect was observed in the tBLM mimicking a neuronal membrane (Figure 4D). The peak continuously shifted toward higher defect density values with increasing Aβ42 incubation time. Following 30 min of membrane incubation with Aβ42 oligomers, two distinct peaks emerged in the probability curve, indicating the development of a heterogeneous distribution of defects in lipid membranes. Integration of the probability curves allows an estimation of the expected average defect density. This data indicated that 10 min of exposure to Aβ42 increased the average defect density in tBLMs from 0.24 to 0.70 μm^–2^, with a further increase to 2.53 μm^–2^ after 30 min (Table S2). In addition, the maximum observed in the low-defect-density region of the distribution curve for the 30 min Aβ42-treated tBLM falls within the range characteristic of naturally occurring defects in lipid membranes. This feature of the distribution curve likely reflects a partial separation between regions containing naturally occurring lipid bilayer defects and those containing a mixture of Aβ42-induced and natural defects in tBLMs.^34^

**Figure 4:**
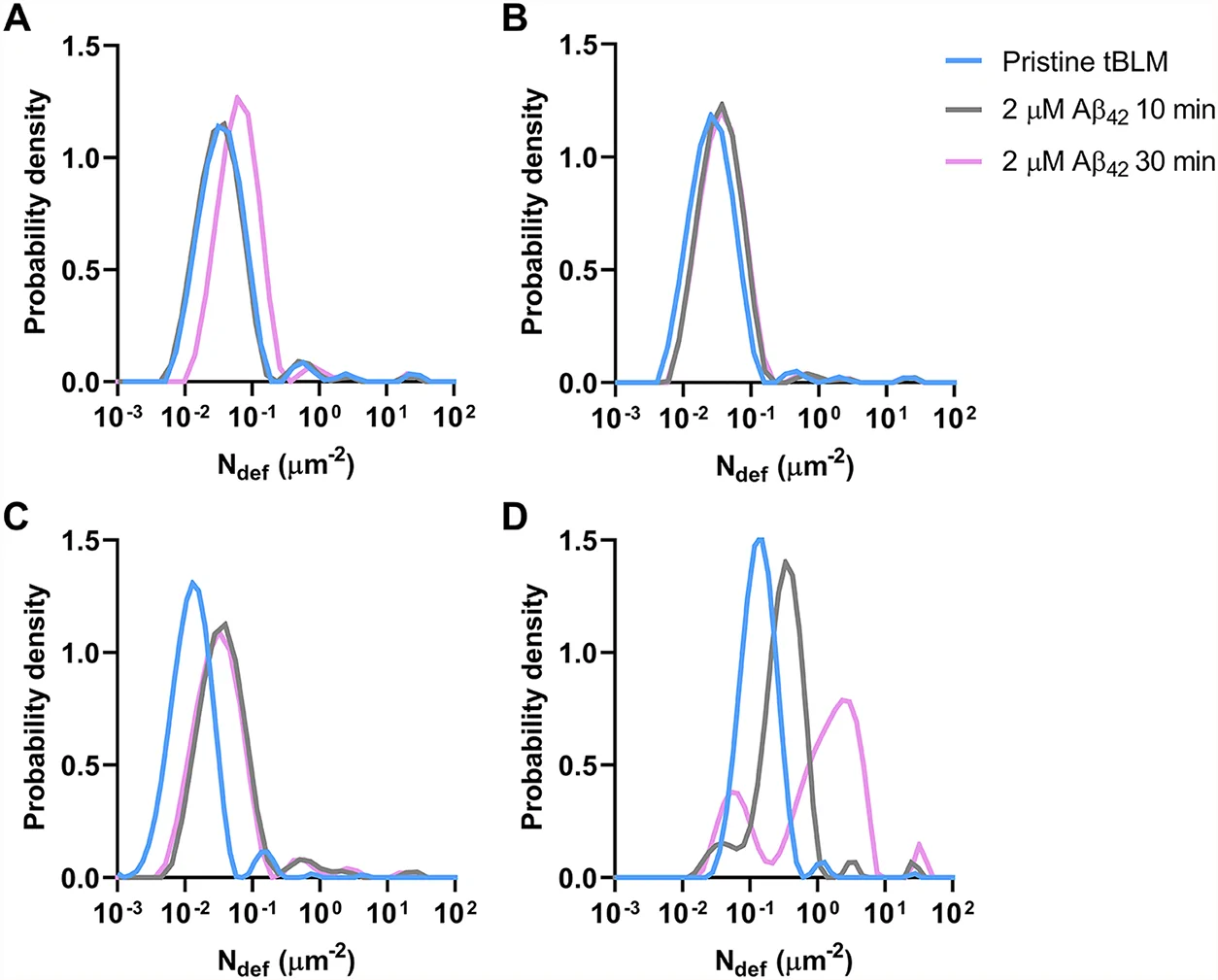
Fitted distributions of defect densities in tBLMs composed of (A) DOPC/Chol/GM1 (55/40/5), (B) DOPC/Chol/SM (45/40/15), (C) DOPC/Chol/DOPE (50/40/10), and (D) the neuronal outer leaflet-mimicking tBLM DOPC/Chol/SM/DOPE/GM1 (30/40/15/10/5), before and after treatment with Aβ42.

EIS measurements revealed that Aβ42 induces significant membrane perturbations only in the complex, five-component lipid environment, whereas simpler bilayers remain largely stable and impermeable. The specific lipid combination likely promotes stronger or more destabilizing interactions with Aβ42 oligomers. Notably, the presence of the DOPC/Chol/SM/DOPE/GM1 lipid mixture also reduces the rate of Aβ42 aggregation (Figure 1), potentially prolonging the lifetime of toxic intermediate species. This extended persistence may enhance membrane disruption and account for the observed EIS response. Although the exact mechanism remains unclear, our results indicate that lipidic composition strongly influences the ability of Aβ42 oligomers to disrupt membrane integrity. The higher average initial conductivity of the five-component membrane may reflect differences in the size or distribution of naturally occurring defects (Figure 3B). These defects may arise from lipid-packing mismatches between lipids with different molecular shapes (e.g., cylindrical vs conical) in membranes.^41^ In addition, irregularities in the supporting solid surface may further contribute to the formation of natural defects in pristine tBLMs.^34^ Our data is consistent with the idea that Aβ42 oligomers more readily perturb membranes with a higher packing defect density. This interpretation aligns with reports for other amyloidogenic proteins (α-synuclein), which have been shown to preferentially associate with more loosely packed bilayers.^42^ Packing defects create gaps between lipid headgroups, lowering the energetic barrier for insertion of hydrophobic protein residues. In such environments, minimal membrane rearrangement is needed to accommodate the peptide, making insertion and the resulting disruption more favorable.^43^

### 2.6 Microviscosity Changes in Aβ42-Treated tBLMs

EIS measurments revealed that Aβ42 increased tBLM conductivity only in the DOPC/Chol/SM/DOPE/GM1 membrane composition, whereas the other lipid compositions remained largely unaffected. This compositional dependence suggests that Aβ42–membrane interactions may not be limited to the formation of discrete conductive defects. In membranes exhibiting increased conductivity, additional perturbations of bilayer physical properties may accompany or precede the observed electrical response. To examine whether Aβ42 alters membrane biophysical properties beyond those detectable by impedance measurements, fluorescence lifetime imaging microscopy (FLIM) was performed on tBLMs stained with the molecular rotor BODIPY-PM. The fluorescence lifetime of BODIPY-PM reports on local membrane microviscosity, providing sensitivity to changes in lipid packing and molecular mobility within the bilayer.^44^

Treatment of tBLMs, containing DOPC/Chol/GM1 with Aβ42 oligomers, increased the intensity-weighted fluorescence lifetime of BODIPY-PM from 1600 to 2100 ps (Figure 5A,B), corresponding to an increase in microviscosity from 75 to 160 cP. We note that tBLMs often display stochastic heterogeneities in lipid order, likely arising from uneven spatial lipid distributions in the lipid membrane.^45^ Interestingly, Aβ42 oligomer exposure gradually homogenized the lipid bilayer in a time-dependent manner (Figure 5B). The observed increase in microviscosity suggests that Aβ42 oligomers enhance lipid ordering and restrict lipid mobility. Similarly, in DOPC/Chol/SM tBLMs, fluorescence lifetime increased from 1350 to 1650 ps, corresponding to a rise in microviscosity from 60 to 80 cP, indicating peptide-induced bilayer ordering (Figure 5C,D).

**Figure 5:**
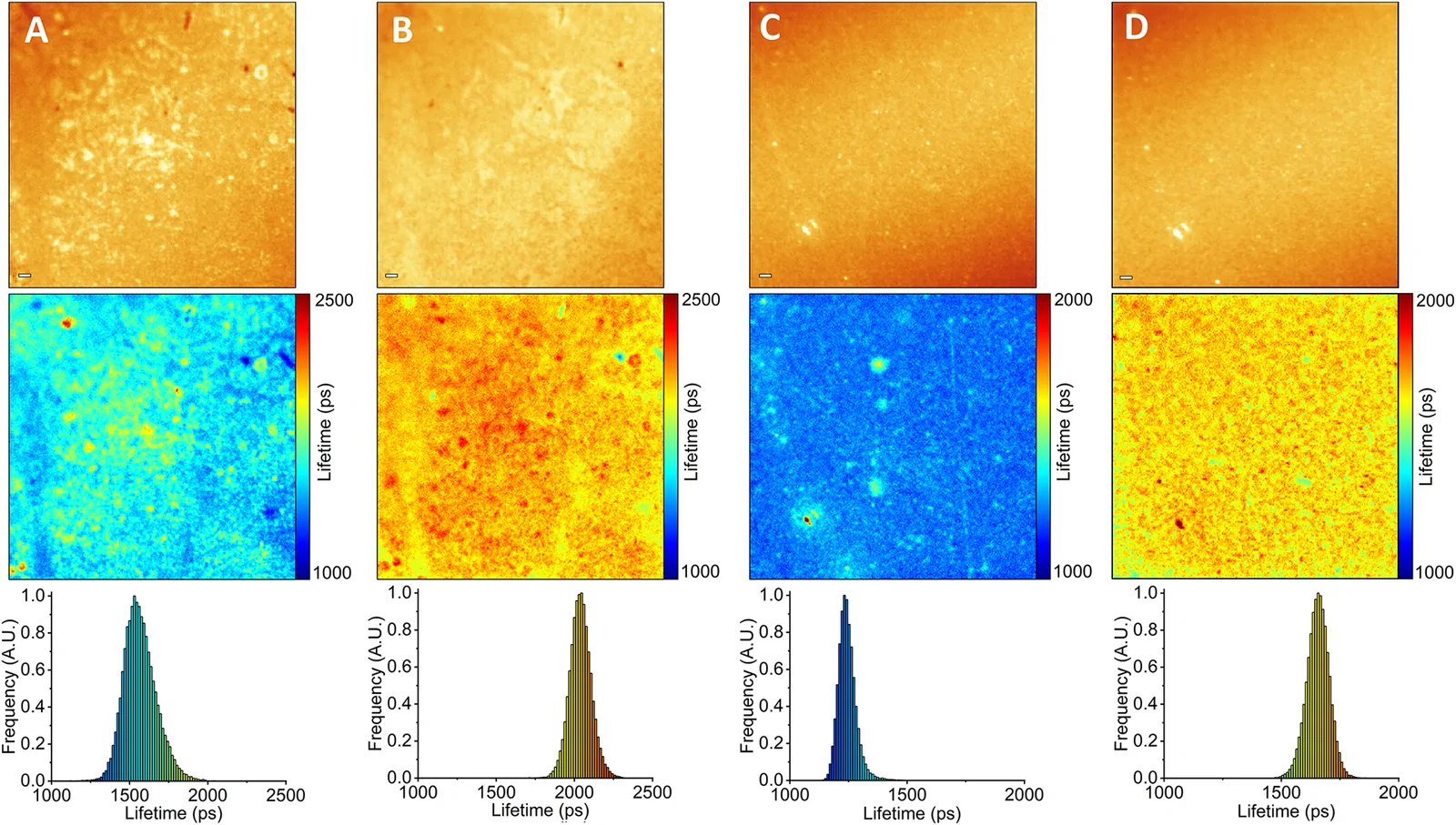
FLIM images of BODIPY-PM in DOPC/Chol/GM1 (55/40/5) tBLM (A) before and (B) after Aβ42 oligomer treatment. FLIM images of BODIPY-PM in DOPC/Chol/SM (45/40/15) tBLM (C) before and (D) after addition of 2 μM Aβ42 oligomers. The top panel shows images of fluorescence intensity. FLIM images are shown in the middle panel. The corresponding lifetime histograms are shown in the bottom panel. Scale bars are 1 μm.

Despite these changes in microviscosity, no substantial increase in membrane conductance was detected in either composition. Thus, the observed changes in microviscosity were not accompanied by clearly measurable changes in membrane permeability. One possible explanation is that Aβ42 associates predominantly with the membrane interface in these systems. This observation is consistent with previous studies that support an interfacial binding mode. In GM1-containing membranes, the oligosaccharide headgroup, particularly the sialic acid residue, may provide sites for electrostatic and hydrogen-bonding interactions with Aβ.^46,47^ Similarly, Aβ has been reported to associate with sphingomyelin-rich membrane environments while remaining predominantly at or near the membrane interface.^48^ Such interactions could restrict local lipid mobility without causing extensive penetration into, or disruption of the hydrophobic core.

In tBLMs containing DOPC/Chol/DOPE, FLIM maps and lifetime distributions were unchanged upon Aβ42 exposure: the intensity-weighted average lifetime remained 1200 ps (53 cP) with comparable dispersion, and no new long or short lifetime domains emerged (Figure 6A,B). Together with the unchanged membrane conductance observed in the EIS measurements, these results suggest that Aβ42 oligomers neither alter membrane fluidity nor promote defect formation in lipid membranes, containing DOPC/Chol/DOPE, which exhibit homogeneous microviscosity. A markedly different response was observed in the neuronal outer leaflet-mimicking DOPC/Chol/SM/DOPE/GM1 tBLM. Before Aβ42 addition, this membrane exhibited pronounced lateral heterogeneity, with regions of higher BODIPY-PM lifetime relative to the surrounding membrane. Following exposure to Aβ42 oligomers, the intensity-weighted fluorescence lifetime decreased from 1900 to 1600 ps (130 to 75 cP) (Figure 6C,D). This decrease was accompanied by reduced contrast between regions of different lifetime and by a significant increase in membrane conductance detected by EIS. The combined FLIM and EIS results therefore indicate that Aβ42-induced permeabilization in the five-component membrane is associated with the disruption of lipid packing and partial homogenization of the initially heterogeneous microviscosity landscape.

**Figure 6:**
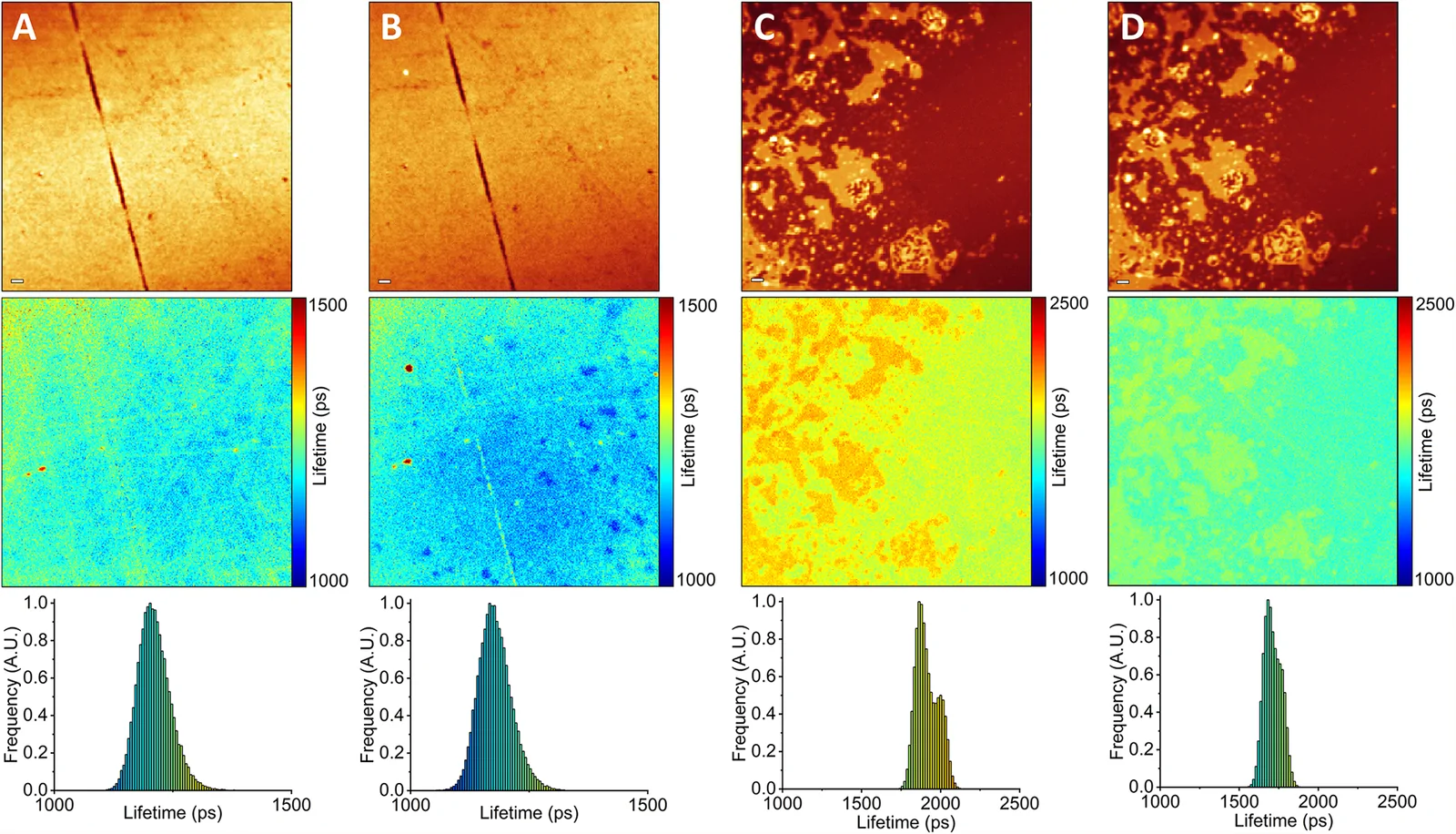
FLIM images of BODIPY-PM in DOPC/Chol/DOPE (50/40/10) tBLM (A) before and (B) after Aβ42 treatment. FLIM images of BODIPY-PM in DOPC/Chol/SM/DOPE/GM1 (30/40/15/10/5) tBLM (C) before and (D) after addition of 2 μM Aβ42. The top panel shows images of fluorescence intensity. FLIM images are shown in the middle panel. The corresponding lifetime histograms are shown in the bottom panel. Scale bars are 1 μm.

The five-component lipid mixture has physicochemical properties that favor lateral heterogeneity in lipid packing and possibly support raft-related organization as it contains cholesterol, sphingomyelin, and GM1. Related model membranes containing DOPC, sphingomyelin, cholesterol, and GM1 have been shown to form GM1-enrinched domains, while phosphatidylcholine/sphingomyelin/cholesterol systems can exhibit coexistance of liquid ordered and liquid disordered (*L*_o_/*L*_d_) phases.^49–53^ However, phase behavior depends strongly on lipid molar ratio, membrane architecture, and experimental conditions.^52–54^ Therefore, the observed heterogeneity should not be interpreted as direct evidence of classical lipid rafts or well-defined *L*_o_/*L*_d_ phase coexistence. Based on the FLIM images, the optically resolved regions of different microviscosity had lateral dimensions of 0.3–2 μm, with some larger irregular regions extending to 2–3 μm. Smaller nanodomains may also be present, but they are below the spatial resolution of the present imaging approach. BODIPY-PM reports local microviscosity rather than regions of specific lipids. Although GM1 commonly associates with cholesterol- and sphingomyelin-rich ordered environments in model systems, its distribution depends strongly on membrane composition, GM1 concentration, and cholesterol content.^49–52^ GM1 has been shown to form clusters in sphingomyelin- and cholesterol-containing membranes^49^ and to influence the size distribution and boundary properties of ordered domains in a cholesterol-dependent manner.^51^ The interpretation of the observed heterogeneity must also account for the tethered architecture of the membranes. tBLMs provide several important advantages, including high mechanical stability, controlled lipid composition, strong electrical sealing, and compatibility with both electrochemical impedance spectroscopy and fluorescence-based microscopy.^45,55–59^ These properties enable time-resolved measurements of membrane characteristics, including conductance, defect formation, and local microviscosity, within the same model system. However, coupling of the bilayer to a planar support may also influence membrane organization. Nonuniform distribution of tethering molecules, variations in tether density, organization of the underlying self-assembled monolayer, coupling of the proximal leaflet to the tethering layer, and restricted lateral lipid mobility may promote or preserve local differences in lipid packing and membrane order in tBLMs.^55,60–62^ The planar support may additionally restrict the accommodation of local bilayer-thickness mismatch. In free-standing membranes, differences arising from lipid chain length or lipid order can be partially compensated by membrane bending and lateral lipid redistribution.^63,64^ In tBLMs, coupling of the proximal leaflet to the tethering layer and reduced lateral lipid mobility may constrain these adjustments,^54,55,60,65^ allowing local variations in bilayer thickness and lipid packing to persist. This interpretation is supported by our observation that other membrane model systems with the same lipid composition did not exhibit comparable heterogeneity (Figure S6). Consistent with this interpretation, Shenoy et al. demonstrated that tBLM homogeneity and lipid dynamics strongly depend on tether architecture and preparation conditions, and that lateral heterogeneity in tBLMs does not necessarily correspond to distinct classical lipid phases.^66^ Overall, these findings suggest that the observed domain-like heterogeneity may be substrate-dependent and determined by the specific architecture of the tBLM.

Although our data do not allow direct determination of the precise membrane partitioning of Aβ42, we speculate that the distinct response of the DOPC/Chol/SM/DOPE/GM1 tBLM may reflect preferential association of Aβ42 oligomers with regions of heterogeneous lateral packing. Notably, the five-component composition exhibited the highest initial conductance (Figure 3B), indicating lower electrical barrier properties. Heterogeneity in microviscosity may create interfaces between membrane regions with different lipid-packing properties. Differences in lipid order and bilayer thickness across these interfaces may produce local packing discontinuities and increased mismatch.^64,67^ Such structurally perturbed regions could favor the association and partial insertion of Aβ42 oligomers into the membrane. Preferential interaction with these heterogeneous regions could contribute to the decrease in overall microviscosity and the increase in membrane conductance observed in the DOPC/Chol/SM/DOPE/GM1 tBLM. Local disruption of lipid packing could also reduce the contrast between regions of higher and lower microviscosity, resulting in the more homogeneous membrane appearance observed by FLIM. Similar boundary-associated behavior has been reported for several antimicrobial and amphipathic peptides, some of which preferentially localize at interfaces between *L*_o_ and *L*_d_ phases and alter domain-boundary organization.^68–70^ Given that lipid rafts are characterized by relatively tightly packed lipids and serve as platforms for the organization of membrane receptors and signaling proteins, Aβ42-induced alterations in raft-like membrane organization and microviscosity may have important implications for neuronal signaling and synaptic dysfunction.^71^

## 3 Conclusions

This study demonstrates that Aβ42 oligomer-induced membrane perturbation is strongly dependent on lipid composition and the cooperative organization of membrane components. The ThT aggregation assay showed that the five-component DOPC/Chol/SM/DOPE/GM1 vesicles delayed Aβ42 fibrillation at low peptide concentration, suggesting that this complex lipid environment may promote peptide association with the membrane surface and prolong the lifetime of membrane-interacting intermediate species. GM1- and SM-containing DOPC/Chol membranes exhibited increased microviscosity after Aβ42 exposure, indicating peptide–membrane interaction associated with enhanced lipid ordering and reduced lipid mobility. However, these changes were insufficient to induce significant membrane permeability defects. In contrast, the five-component DOPC/Chol/SM/DOPE/GM1 membrane showed a pronounced increase in conductance, loss of its insulating properties, and a time-dependent increase in heterogeneous defect density. Notably, this membrane also exhibited a decrease in microviscosity after Aβ42 treatment, suggesting that peptide-induced membrane permeabilization was accompanied by disruption of lipid packing and partial homogenization of the initially heterogeneous bilayer environment. These findings indicate that extensive Aβ42-induced membrane permeabilization depends on the combined effects of multiple lipid components rather than on the presence of a single Aβ-interacting lipid species. The enhanced susceptibility of the neuronal outer leaflet-mimicking membrane may result from lateral heterogeneity in lipid packing and pre-existing defect-prone regions that facilitate Aβ42 accumulation and subsequent bilayer disruption. While our data support this interpretation, further studies directly probing peptide partitioning and membrane insertion will be necessary to elucidate the underlying molecular mechanisms. Overall, these results support a model in which lipid packing, microviscosity heterogeneity, and cooperative lipid organization jointly determine the membrane vulnerability to Aβ42 oligomer-induced damage.

## 4 Experimental Procedures

### 4.1 Formation of tBLMs

tBLMs were formed on gold (Au)-coated glass substrates. Glass slides (Thermo Fisher Scientific) were deposited with a 10 nm chromium adhesion layer followed by a 100 nm Au layer using a PVD75 system (Kurt J. Lesker) via magnetron sputtering. Au-coated glass slides were subsequently incubated for 3 h in an ethanolic solution containing a mixture of the synthetic lipid anchor 20-tetradecyloxy-3,6,9,12,15,18,22-heptaoxahexatricontane-1-thiol (WC14; synthesized in-house) and β-mercaptoethanol (βME) at a molar ratio of 35:65 (total concentration 0.05 mM). After the spontaneous organization of anchoring and spacer molecules on the surface to form a self-assembled monolayer (SAM), tBLMs were completed by the fusion of multilamellar vesicles (MLVs).^72,73^ For this purpose, vesicle suspensions of four different compositions were prepared: (i) DOPC/Chol/GM1 (molar ratio 55:40:5), (ii) DOPC/Chol/SM (45:40:15), (iii) DOPC/Chol/DOPE (50:40:10), and (iv) DOPC/Chol/SM/DOPE/GM1 (30:40:15:10:5). Stock solutions of lipids in chloroform were mixed in the specified ratios. The chloroform was subsequently evaporated using a nitrogen stream, resulting in the formation of a lipid film on the walls of the glass vial. The dried lipid film was resuspended in a solution containing 0.1 M NaCl and 0.01 M NaH_2_PO_4_, pH 4.5, preheated to 55 °C. An MLV suspension with a total lipid concentration of 1 mM was injected into each vial of the measurement setup (100 μL per vial) and incubated for 1 h. The wells were subsequently washed with phosphate-buffered saline (PBS; 137 mM NaCl, 10 mM Na_2_HPO_4_, 2.7 mM NaH_2_PO_4_, pH 7.4), after which the tBLMs were prepared for measurements. All reagents and solvents were obtained from Sigma-Aldrich. The lipids were obtained from Avanti Polar Lipids.

### 4.2 Formation of LUVs

MLVs were first prepared by following the same lipid hydration protocol used for tethered bilayer lipid membranes. Briefly, the dried lipid film was hydrated in PBS (pH 7.4) to obtain a turbid suspension of the MLVs. The resulting suspension was then ultrasonicated in a water bath for 1 h to further reduce the vesicle size and polydispersity. Finally, large unilamellar vesicles (LUVs) were obtained by extruding the MLV suspension 21 times through 100 nm pore-size polycarbonate membranes using a mini extruder (Avanti Polar Lipids). This procedure yielded a population of vesicles with a relatively uniform size distribution and a unilamellar structure suitable for subsequent experimental applications.

### 4.3 Aβ42 Purification

Aβ42 was purified as previously described.^74^ In short, recombinant Aβ42 expression in *E. coli* BL-21Star was initiated by growing the bacteria in ZYM-5052 autoinduction media^75^ for 16 h. Cells were homogenized and lysed in nondenaturing conditions and centrifuged to remove the supernatant with a large fraction of soluble proteins. This process was repeated three times. Then, the residual cell pellet was homogenized in a denaturing cell lysis buffer solution containing 8 M Urea. After centrifuging at 18000*g* for 30 min, the supernatant was collected and filtered using a 0.45 μm mixed cellulose syringe filter. The target peptide-containing supernatant was diluted 4 times with the loading buffer solution (20 mM Tris, 1 mM EDTA, pH 8.0) and mixed for 30 min with equilibrated DEAE-sepharose. Sorbent was collected using a vacuum filtering system and used for quick peptide purification. Aβ42 peptide was eluted using a NaCl step-gradient; samples were combined, flash frozen, and lyophilized overnight. The resulting Aβ42 peptide powder was dissolved in 10 mM sodium phosphate buffer solution containing 0.2 mM EDTA, 5 M guanidinium thiocyanate (GuSCN) (pH 8.0) and injected into a 16/300 size exclusion column packed with Superdex-75 sepharose equilibrated with SEC buffer solution—10 mM sodium phosphate buffer solution containing 0.2 mM EDTA (pH 8.0). The Aβ42 peptide peak was collected, samples were combined, flash frozen, and lyophilized. The resulting Aβ42 peptide powder was dissolved in 1 mL of 10 mM sodium phosphate buffer solution containing 0.2 mM EDTA, 5 M GuSCN (pH 8.0), and injected into a 10/300 size exclusion column packed with Superdex-75 sepharose, which was previously equilibrated with SEC buffer solution. The Aβ42 peptide peak retention time was 16 min. Aβ42 peptide fractions were collected and used for further experiments.

### 4.4 Aβ42 Aggregation Experiments

The aggregation kinetics of Aβ42 were monitored in PBS (pH 7.4) containing 20 mM thioflavin T (ThT), in the absence and in the presence of LUVs of different lipid compositions (200 μM total lipid). Aliquots (80 μL) of these solutions were dispensed into 96-well, non-binding, flat-bottom, half-area microplates (Corning Inc.) and sealed with adhesive film (Thermo Fisher Scientific). The plates were then placed in a CLARIOstar Plus microplate reader (BMG Labtech), and amyloid fibrillation was monitored by ThT fluorescence using an excitation wavelength of 440 nm and an emission wavelength of 480 nm at room temperature (25 °C), without shaking. ThT fluorescence kinetic data were fitted using a Boltzmann sigmoidal function, and lag times were extracted from the fitted curves.^76^

### 4.5 AFM Imaging

AFM imaging was performed in air using tapping mode on a Dimension Icon microscope (Bruker) equipped with silicon nitride probes FESP-V2 (Bruker). Aβ42 samples (25 μL) were deposited onto freshly cleaved mica functionalized with (3-Aminopropyl)­triethoxysilane (APTES) (Sigma-Aldrich) and incubated for 5 min. The substrates were then extensively rinsed with Milli-Q water and dried under a nitrogen gas stream. Height images were acquired at a resolution of 512 × 512 pixels with a scan rate of 0.8 kHz. AFM images were processed and analyzed using NanoScope Analysis (Bruker) and Gwyddion 2.61 software.^77^

### 4.6 EIS Measurements

Electrochemical impedance measurements were performed using PalmSens4 (PalmSens). EIS spectra were obtained using a 10 mV AC amplitude at a potential of 0 V over a frequency range from 0.5 Hz to 100 kHz. The working electrode was an Au-coated glass slide. As a counter/reference electrode, we used a silver wire. Experiments were carried out in a custom-made setup where a SAM-modified Au-coated glass slide was placed between an aluminum base and an upper part made of Teflon. The upper component contained 14 separate wells for conducting independent experiments. The working surface area of each cell was 0.16 cm^2^. EIS measurements were performed in the PBS (pH 7.4) solution. EIS spectra were obtained from at least four independent measurements of tBLM for each composition.

EIS spectra were fitted as described previously.^31,34^ The submembrane layer parameters were set to a specific resistance of 10^4.5^ Ω and a thickness of 1.8 nm. Values of 2.5 nm for the defect radius and 50 pS for conductance were used throughout the analysis. The regularization parameter was fixed at −0.9 for all fits. The average defect density ⟨*N*_def_⟩ was calculated from the defect-density distribution function according to

1 $$\left\langle N_{\mathrm{def}}\right\rangle =\int_{N_{\min}}^{N_{\max}}P\left(N_{\mathrm{def}}\right)N_{\mathrm{def}}dN_{\mathrm{def}}$$

### 4.7 FLIM

FLIM was performed using a Leica SP8 microscope with a 63x oil immersion objective (HC PL APO, N.A. 1.4, Leica). Fluorescence decay was recorded in the 510–550 nm range with a 488 nm filter-supported white light laser excitation line. FLIM images were acquired at a 256 × 256 pixel resolution and contained at least 1000 counts per pixel at the peak of the decay curve for reliable biexponential fitting. The instrument response function (IRF) was measured by recording the laser reflection signal off a glass coverslip. FLIM images were analyzed with FLIMfit software (v4.6.1, Imperial College London). The biexponential fluorescence decay model with intensity-weighted mean lifetimes was applied for FLIM measurements

2 $$\mathrm{\tau ̃}=\frac{\sum_{i}a_{i}\tau_{i}^{2}}{\sum_{i}a_{i}\tau_{i}}$$

where *a*_*i*_ and τ_*i*_ are the amplitudes and lifetimes of the individual components. The goodness-of-fit parameter (χ^2^) was 1.3 or less for FLIM decays. The tBLMs were stained for 3 min with BODIPY-PM at 1 μM concentration, followed by removal of nonincorporated dye with a PBS wash. BODIPY-PM was synthesized as described previously.^78^

### 4.8 Statistical Analysis

Statistical analysis of the data was conducted using GraphPad Prism version 8.4.3 (GraphPad Software). The Shapiro–Wilk test was used to assess data normality. For Aβ42 aggregation and EIS data, one-way and two-way ANOVA, respectively, were applied, followed by Tukey’s post hoc test, with statistical significance set at *p* < 0.05.

## Supplementary Material



## Notes

Dynamic light scattering data for liposome hydrodynamic diameters, electrochemical impedance spectroscopy data for tBLM formation and stability, concentration-dependent tBLM responses to Aβ42 oligomers, fitted EIS spectra, and defect density analysis of tBLMs with different lipid compositions (PDF)
