# Supplementary material for: Impact of Lipid Composition and Synergistic Effect on Aβ42 Oligomer-Induced Changes in Membrane Conductance and Microviscosity
Source: ACS Chem Neurosci. 2026 Aug 31;17(18):3306–17. doi: 10.1021/acschemneuro.6c00244 (PMC13587654; doi:10.1021/acschemneuro.6c00244)
Supplement: Supplementary file 1 [file cn6c00244_si_001.pdf]

Supporting Information

Impact of Lipid Composition and Synergistic Effect on Aβ42 Oligomer-Induced Changes in Membrane Conductance and Microviscosity

Evelina Jankaityte[1*], Artūras Polita[1], Andrius Sakalauskas[2], Rimgailė Tamulytė[1], Vytautas Smirnovas[2], Rima Budvytyte[1]

[1] *Vilnius University, Life Sciences Center, Institute of Biochemistry, Sauletekio av. 7, Vilnius LT-10257, Lithuania*

[2] *Vilnius University, Life Sciences Center, Institute of Biotechnology, Sauletekio av. 7, Vilnius LT-10257, Lithuania*

*Corresponding author: evelina.jankaityte@bchi.stud.vu.lt

Hydrodynamic diameters of liposomes with different lipid compositions were measured by dynamic light scattering before and after 2 h incubation with 2 µM Aβ42 oligomers (Table S1). Initial Z-average diameters ranged from 125 to 154 nm, while values after incubation ranged from 138 to 191 nm across the tested compositions.

Table S1. Mean Z-average hydrodynamic diameter of liposomes of different lipid compositions measured before and after 2 h incubation with 2 µM Aβ42 oligomers. Dynamic light scattering data (n=3).

Lipid composition	Z-average ± SD (d. nm)	
	**Initial**	**After 2 h with Aβ42**
**DOPC/Chol/SM**	144.7 ± 38.06	164 ± 87.61
**DOPC/Chol/DOPE**	154.1 ± 52.44	191 ± 124.1
**DOPC/Chol/GM1**	129.9 ± 60.48	139 ± 51.79
**DOPC/Chol/SM/DOPE/GM1**	125 ± 48.02	137.8 ± 57.94

The formation and stability of the tethered bilayer lipid membranes (tBLMs) used in the experiments are shown in Fig. S1 and S2, respectively. The lowest Aβ42 oligomer concentration that produced a reliably detectable change in the impedance spectra was 2 µM, as determined from a series of electrochemical impedance spectroscopy (EIS) measurements (Fig. S3).

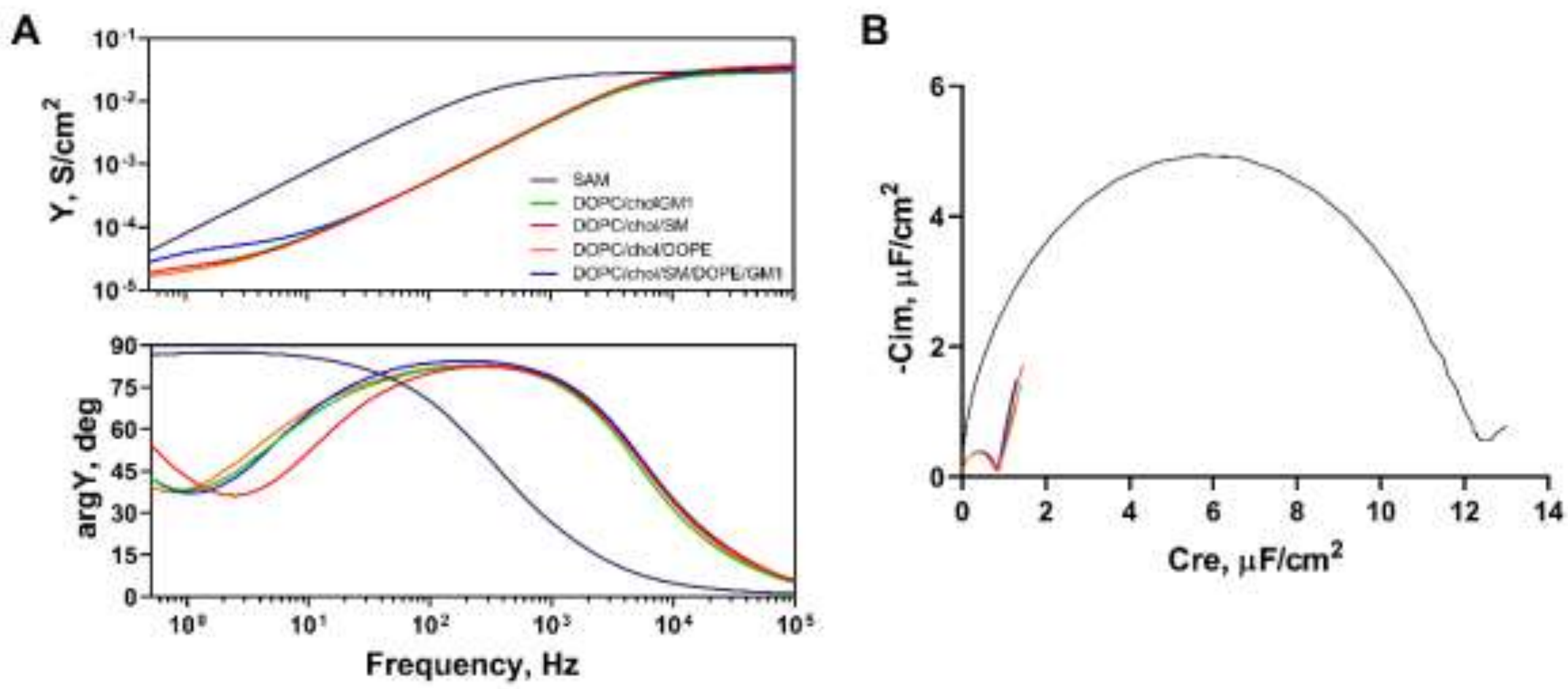

Figure S1. EIS characterization of the self-assembled monolayer (SAM) and formed tBLMs with different lipid compositions. (A) Bode plots. (B) Complex capacitance plot. The black traces correspond to the SAM, whereas the colored traces represent the formed tBLMs, with each color indicating the lipid composition shown in the legend

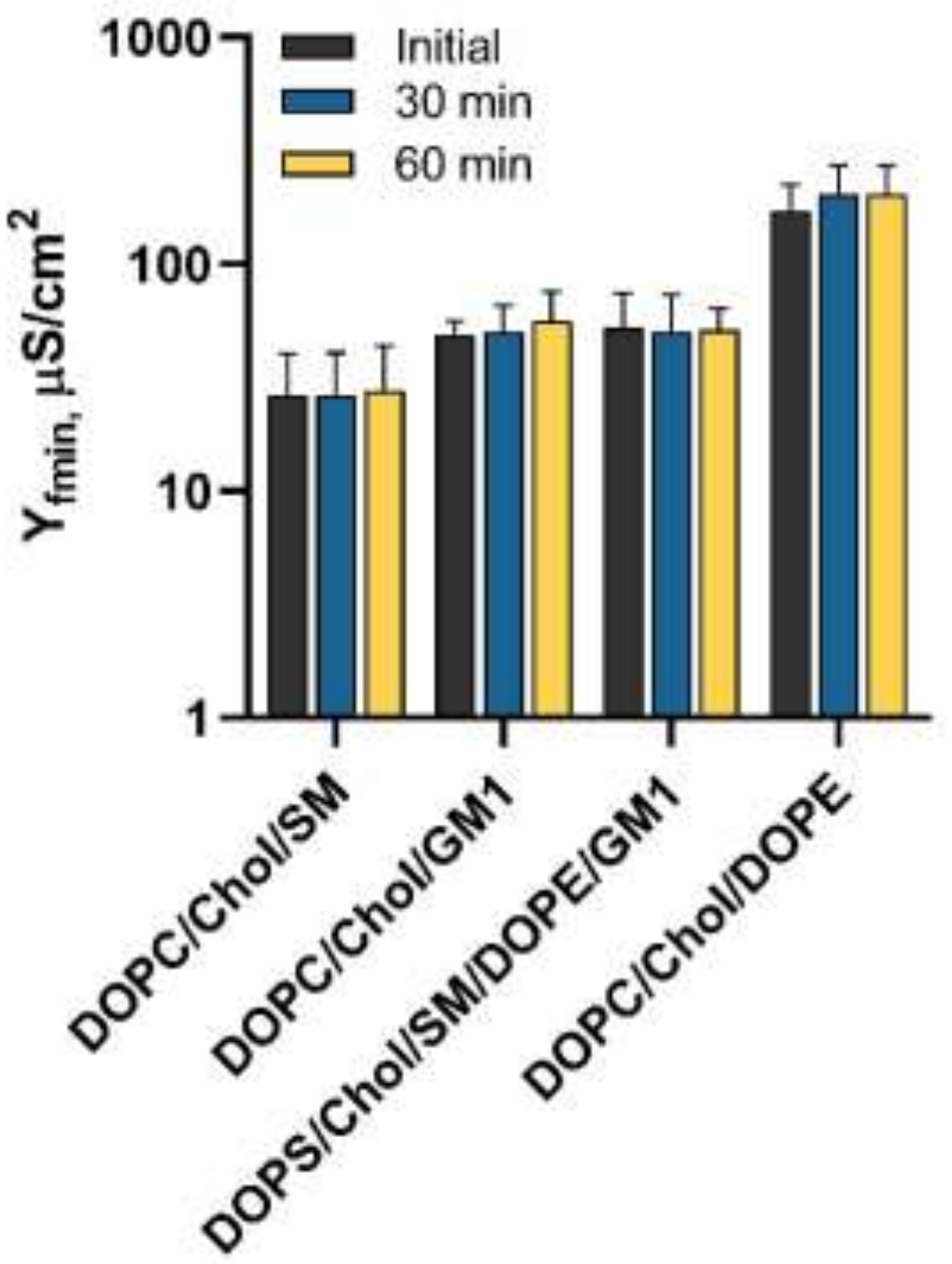

Figure S2. Admittance values measured at the phase angle minimum (f_{min}) for formed tBLMs with different lipid compositions over 60 min.

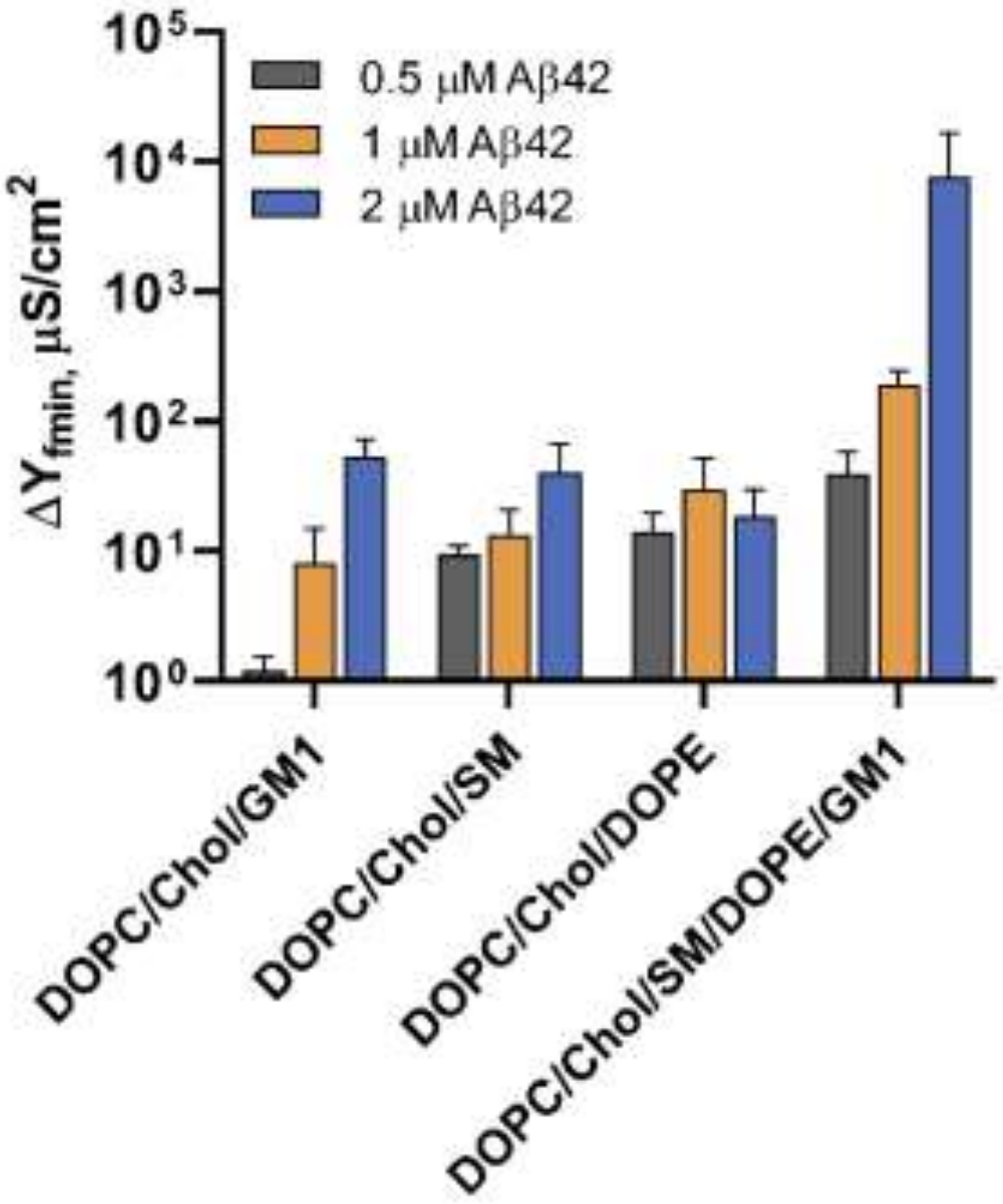

Figure S3. Concentration-dependent changes in the minimum admittance (ΔY_{fmin}) of formed tBLMs with different lipid compositions in response to Aβ42 oligomers. ΔY_{fmin} is the difference between the values measured before and after Aβ42 exposure.

No major changes were observed in the impedance response of tBLMs composed of DOPC/Chol/GM1, DOPC/Chol/SM, or DOPC/Chol/DOPE following exposure to Aβ42 oligomers (Fig. S4 and S5).

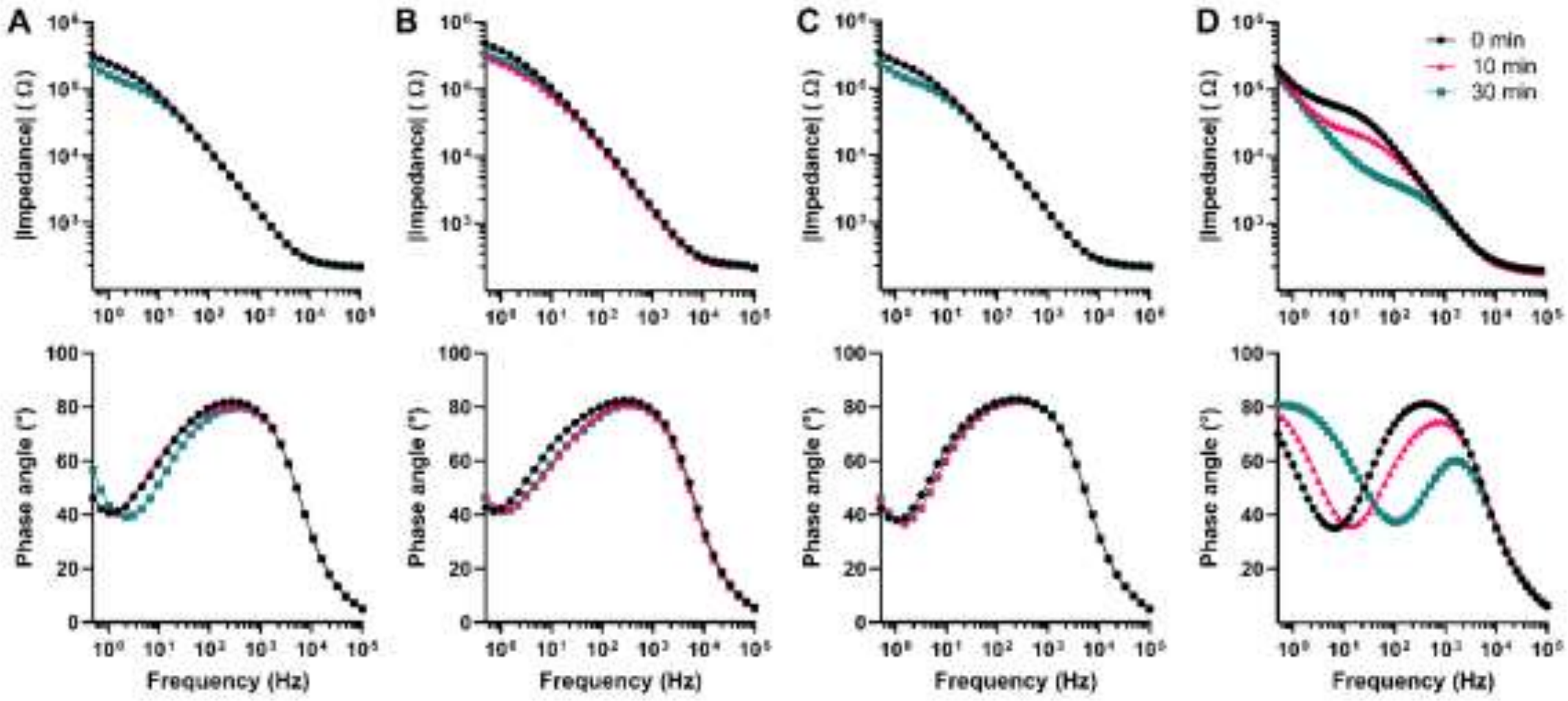

Figure S4. Representative EIS Bode plots of tBLMs composed of (A) DOPC/Chol/GM1, (B) DOPC/Chol/SM, (C) DOPC/Chol/DOPE, and (D) DOPC/Chol/SM/DOPE/GM1 recorded before (0 min) and after 10 and 30 min of incubation with 2 µM Aβ42 oligomers. The upper and lower panels show the impedance magnitude and phase angle, respectively.

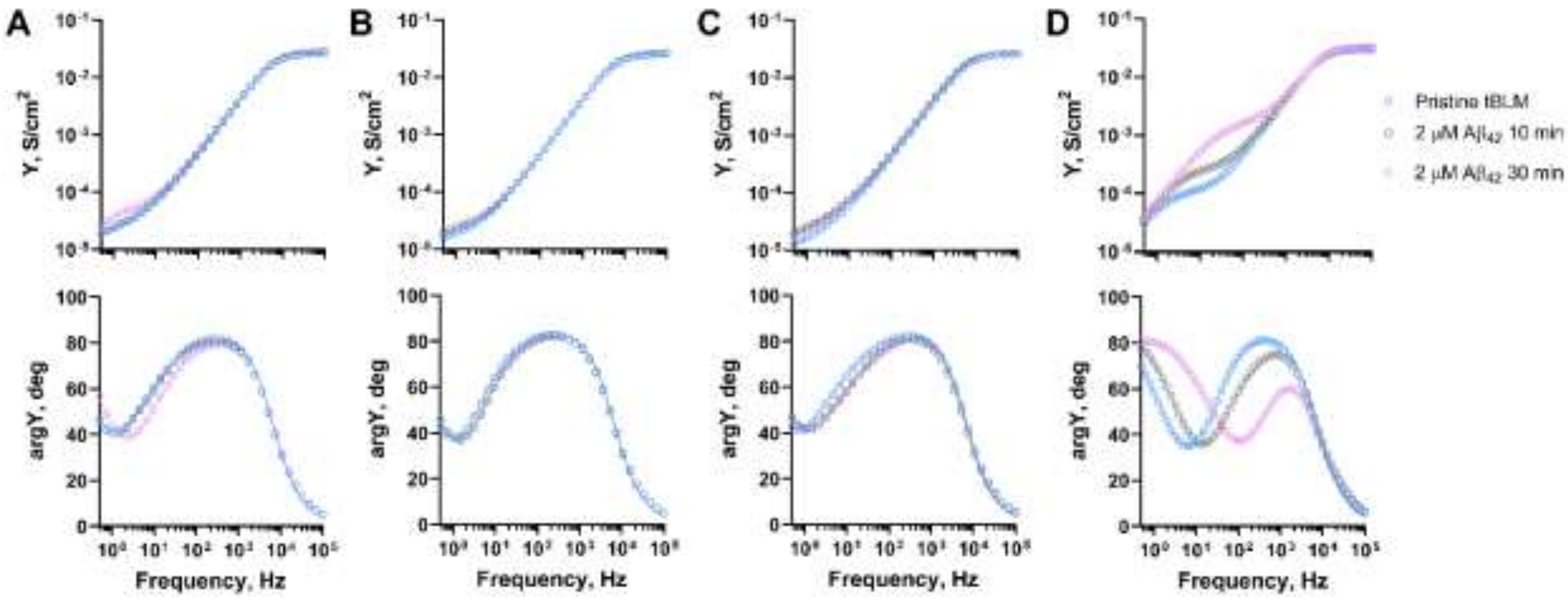

Figure S5. Fitted EIS spectra of different tBLMs. (A) DOPC/chol/ GM1 (B) DOPC/chol/SM (C) DOPC/chol/DOPE (D) DOPC/chol/SM/DOPE/GM1 bilayers upon exposure to 2 µM of Aβ42 oligomers. The upper panel of graph displays a plot of *Y* vs. frequency, while the lower panels present a plot of arg*Y* vs. frequency.

Calculated local defect density (N_{def}) distributions of pristine membranes exhibited a single peak profile, consistent with naturally occurring, monodisperse defects. Upon Aβ42 addition, membranes composed of DOPC/Chol with GM1, SM, or DOPE showed negligible changes in defect density, whereas an apparent effect was observed for the tBLM mimicking a neuronal membrane (Table S2).

Table S2: Time-dependent changes in average defect density in tBLMs with different lipid compositions after exposure to 2 µM Aβ42 oligomers.

tBLM composition	Aβ42-induced defect density in tBLM (µm^{-2})		
	**Initial tBLM**	**After 10 min**	**After 30 min**
**DOPC/Chol/SM**	0.46	0.49	0.50
**DOPC/Chol/DOPE**	0.07	0.63	0.50
**DOPC/Chol/GM1**	0.20	0.18	0.27
**DOPC/Chol/SM/DOPE/GM1**	0.24	0.70	2.53

To address whether the apparent domains observed in DOPC/Chol/SM/DOPE/GM1 tBLMs represent intrinsic L_o/L_d phase separation, we prepared the same lipid composition using alternative membrane platforms (Fig. S6). GUVs of this composition did not exhibit clear micrometer-scale phase separation, while supported lipid bilayers showed only weak heterogeneity in the fluorescence lifetime maps. In contrast, the tBLM system displayed substantially stronger lateral heterogeneity. These observations suggest that the heterogeneity detected in tBLMs is likely promoted or amplified by the tethered membrane architecture.

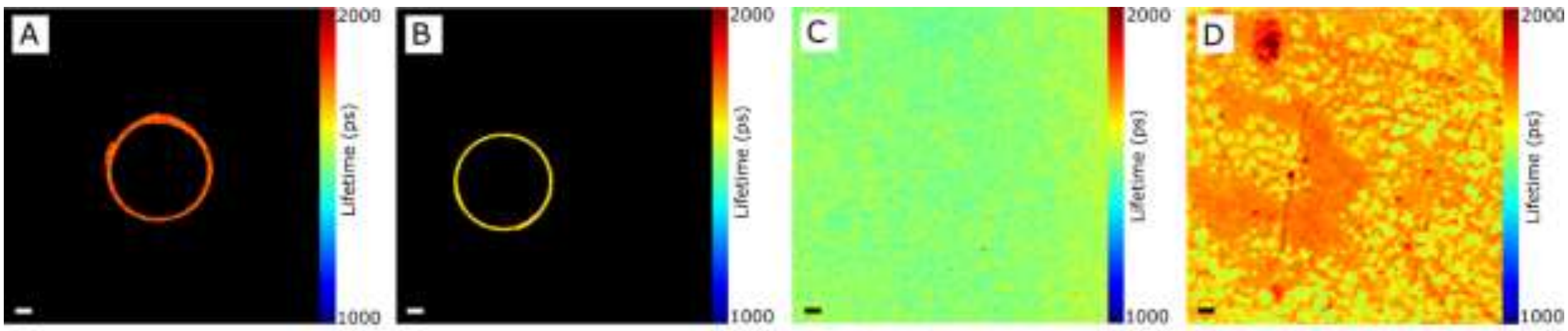

Figure S6. Representative fluorescence lifetime maps of DOPC/Chol/SM/DOPE/GM1 membranes prepared in different membrane model systems. Images of (A, B) giant unilamellar vesicles (GUVs), scale bars 3 µm, (C) supported lipid bilayer (SLB), scale bar 1 µm, and (D) tethered bilayer lipid membrane (tBLM), scale bar 1 µm.
